# American Medical Association, Section on Stomatology

**Published:** 1902-05

**Authors:** 


### AMERICAN MEDICAL ASSOCIATION, SECTION ON STOMATOLOGY.

DR. GEORGE V. I. BROWN, of Milwaukee, read a paper on "Surgical Correction of Malformation and Speech Defects due to or associated with Harelip and Cleft Palate."

(For Dr. Brown's paper, see page 301.)

### DISCUSSION.

*Dr. James G. Kiernan,* Chicago.—There was one point incidentally brought out in this paper which was new and which explains why the cleft palate has been found apparently not hereditary. Dr. Brown alluded to the fact of danger of bacterial infection in these cases. At one time I was a health officer, and found that a large number of cases had been so infected and died in infancy. With regard to the statement made by Dr. Brown about the speech, not only is it perfectly justifiable from the stand-point of neurology, without dealing with the question of details, but to a certain extent it applies to a certain class of deaf mutes and to a class of idiots who become deaf mutes on account of the lack of trying to follow pretty much along the same lines. I think from that standpoint the paper deserves particular attention. To a certain extent there is a number of individuals with cleft palates who are a survival of conditions existing quite early in development and some comparatively late. There are human beings who are in a condition similar to some of the lower apes, and this gives the title to this number. With regard to the nasal septum, that is like the survival in a degree of the jowl-like condition which is presented quite early in the development. The human would not develop alone the regular face and nose, but he would have with the nose and face a jowl-like appearance which is found among the lower apes.

I think the paper deserves special attention because it was so practical, not only in the general treatment of these conditions, but in the surgical treatment. A large number of these cases are mentally defective, and that fact should be taken into account in dealing with them.

*Dr. D. Baldwin Wylie,* Milwaukee, Wis.—I do not know that I should be allowed to speak before this Section. I am here on account of the great interest I feel in the paper that has just been read by Dr. Brown, and largely because I have had opportunity to observe the work. The results obtained by Dr. Brown in this work are really worthy of the most earnest consideration. The matter of speech that the doctor has taken up in a somewhat exhaustive manner is one of considerable interest also. The doctor, with his usual modesty, has given himself little credit for it by overlooking or neglecting, or purposely keeping from view, the fact that persons' ears are different. The cleft palate, as you all know, is, as a rule, associated with other forms of arrested development, and almost always there is more or less defect of the hearing apparatus associated with it. Now, we all know there are human beings, to all intents and purposes normal in development, who could not, if their life depended upon it, learn so simple an air as Yankee Doodle. This is simply because they have not the perceptive power of hearing necessary for it. Now, if a perfectly developed person, so far as we can see, is defective in the matter of imitation of sound, which, of course, is only a secondary result of perfect hearing, it seems to me to be so very patent that it hardly needs mentioning, that a person who is unquestionably and evidently defective in the matter of development could very easily have a defect of the hearing apparatus that would make it impossible for him to receive musical sounds or other sounds with sufficient accuracy to reproduce them with any resemblance whatever to the original sound. I know by personal experience coming from an acquaintance with Dr. Brown that he has felt at times a little despondent because he cannot get better results in the matter of speech, and I merely raise this point because it seems to me that even though, as in this case, he gets no speech at all, he has certainly accomplished something that, while perhaps not wonderful, is certainly worthy of the effort of any man. This little patient is an example, and points you very strongly to the subject I have just raised. He is absolutely defective in the matter of hearing, and if he is able to reproduce violin sounds that are conveyed to his brain-centre through the medium of bone conduction, it is simply an unusually developed ability to interpret sound-vibrations through other sources than that of the auditory nerve.

*Dr. George T. Carpenter,* Chicago.—I was very much pleased with the paper and with the illustrations. I am sorry that the models do not show the conditions a little farther back, so as to give the anterior pillars and also the uvula, which are the essential points in the reproduction of the voice. The doctor in his remarks spoke of the patient with simply a cleft in the soft palate, the voice being much worse than where the cleft was clear through the hard palate and extending on each side of the intermaxillary bone. My experience has been that that is the common condition. In the large clefts with the double harelip frequently the voice is very much better than where there is a partial cleft of the soft palate. Some of the worst cases I have had, so far as the voice is concerned, have been from the smaller defects in the soft palate. I attribute the reason for this to an intent on the part of nature to overcome those larger clefts which is done in the majority of cases of hypertrophy of the inferior turbinated bones, and in that way control to a certain extent the voice. The pillars hang heavily on the sides from the large clefts, and the patient has more control than one with simply a slight defect. I believe these operations should be performed as soon as the child can well stand an anæsthetic. The great trouble has been with the lack of power to stand the anæsthetic, and we must wait until the child is sufficiently robust and the deciduous teeth have taken their places in the arch, although if the child were in good condition I would prefer operating,—the younger the better. In cases in advanced life or young adults I have had very poor success with the voice with surgical operations. The doctor speaks of this slow method of drawing the parts together. I understand from the paper that he draws the parts together, getting all the margins approximate and then dressing the margins and making his union, but the great difficulty I have found has been to overcome the tension. I can get union by dividing the muscles on either side and frequently raising the periosteum and raising the parts of older patients. You have hard work to make the parts approximate on account of resistance, and by raising the periosteum and sliding the margins together, and then making incisions to overcome the tension, you can get union, but even then I have had tension so the voice was not improved, and I have one case that I have operated on twice and afterwards made an incision and put on an obturator. In persons fifteen years of age or over it has been my experience that the obturator will improve the voice more than a dentist's plate, and it has been a difficulty to secure sufficient tissue to accomplish the adjustment made to the true size.

In reference to education, the whole thing is a matter of education on the part of the patient. A patient with a cleft palate can be educated so as to speak almost distinctly with the cleft existing, if he will take the time. It is a good deal like stammering. The habit is formed and they forget themselves, but if sufficient time be taken not to speak faster than the tongue can follow, the habit can be cured entirely. Although the operation for cleft palate is a great success before the voice is acquired, I think the cases are comparatively rare where we get good voice-sounds. Anatomically they are much improved with the obturator, but there is much more to go through with, and the ultimate outcome is not so good.

*Dr. G. V. I. Brown,* Milwaukee, Wis.—Perhaps I did not make the point clear. The point of resistance is over the bone. The work is done gradually. The borders are finished off with the surgical engine and put in place. Then, after adjusting my appliance, I made a powerful clamp, sprung it right over the bone, and bent it over until I brought it together. During this process of union I continued the screw right along and got it over as much as I could, and then afterwards narrowed the cleft at the back by tightening the screw.

I wish to say, in regard to the matter of speech, I am not speaking of any speech you get from the obturator, but of getting rid of the nasal sound. I think the chief difficulty in those clefts of the soft palate is not so much due to the cleft as to the fact that in those cases we have the nasopharynx filled with adenoid vegetations. I operated on a little fellow who had only a slight cleft, and came near losing the patient. I discovered that an operation had been attempted before, but without success, as he was only half-anæsthetized, for the reason that no one had been paying attention to the fact that the pharynx was filled with vegetations. The inability to close that space is not so great as the inability to close it perfectly.

